# Addressing health literacy in patient decision aids

**DOI:** 10.1186/1472-6947-13-S2-S10

**Published:** 2013-11-29

**Authors:** Kirsten J McCaffery, Margaret Holmes-Rovner, Sian K Smith, David Rovner, Don Nutbeam, Marla L Clayman, Karen Kelly-Blake, Michael S Wolf, Stacey L Sheridan

**Affiliations:** 1 Sydney School of Public Health, Sydney Medical School, Edward Ford Building (A27), The University of Sydney, NSW 2006, Australia; 2 Center for Ethics and Humanities in the Life Sciences, College of Human Medicine, Michigan State University, East Fee Hall, 965 Fee Road Rm C-203, East Lansing, Michigan 48824, USA; 3 Psychosocial Research Group, Prince of Wales Clinical School, Faculty of Medicine, University of New South Wales, Prince of Wales Hospital, Dickenson Building, Level 3, Randwick, NSW 2031, Australia; 4 Office of the Vice-Chancellor, University of Southampton, University Road, Southampton SO17 1BJ, UK; 5 Division of General Internal Medicine, Feinberg School of Medicine, Northwestern University, 750 North Lake Shore Drive, 10th Floor, Chicago, Illinois 60611, USA; 6 Division of General Medicine and Clinical Epidemiology, The University of North Carolina at Chapel Hill, 5039 Old Clinic Building, CB#7110, Chapel Hill, North Carolina 27599, USA

## Abstract

**Background:**

Effective use of a patient decision aid (PtDA) can be affected by the user’s health literacy and the PtDA’s characteristics. Systematic reviews of the relevant literature can guide PtDA developers to attend to the health literacy needs of patients. The reviews reported here aimed to assess:

1. a) the effects of health literacy / numeracy on selected decision-making outcomes, and b) the effects of interventions designed to mitigate the influence of lower health literacy on decision-making outcomes, and

2. the extent to which existing PtDAs a) account for health literacy, and b) are tested in lower health literacy populations.

**Methods:**

We reviewed literature for evidence relevant to these two aims. When high-quality systematic reviews existed, we summarized their evidence. When reviews were unavailable, we conducted our own systematic reviews.

**Results:**

Aim 1: In an existing systematic review of PtDA trials, lower health literacy was associated with lower patient health knowledge (14 of 16 eligible studies). Fourteen studies reported practical design strategies to improve knowledge for lower health literacy patients. In our own systematic review, no studies reported on values clarity *per se*, but in 2 lower health literacy was related to higher decisional uncertainty and regret. Lower health literacy was associated with less desire for involvement in 3 studies, less question-asking in 2, and less patient-centered communication in 4 studies; its effects on other measures of patient involvement were mixed. Only one study assessed the effects of a health literacy intervention on outcomes; it showed that using video to improve the salience of health states reduced decisional uncertainty. Aim 2: In our review of 97 trials, only 3 PtDAs overtly addressed the needs of lower health literacy users. In 90% of trials, user health literacy and readability of the PtDA were not reported. However, increases in knowledge and informed choice were reported in those studies in which health literacy needs were addressed.

**Conclusion:**

Lower health literacy affects key decision-making outcomes, but few existing PtDAs have addressed the needs of lower health literacy users. The specific effects of PtDAs designed to mitigate the influence of low health literacy are unknown. More attention to the needs of patients with lower health literacy is indicated, to ensure that PtDAs are appropriate for lower as well as higher health literacy patients.

## Background

A person’s health literacy status affects their ability to utilise health information and services, and their health outcomes [1]. It is therefore an important potential consideration in patient decision aid (PtDA) development and shared decision making [2,3]. There has been no systematic examination of the effects of health literacy on outcomes relevant to PtDA development or of interventions that might mitigate potential adverse effects of low health literacy in the decision-making context.

Health literacy can be conceptualized in different ways. A simple and common definition is *“the capacity to obtain*, *process*, *and understand basic health information and services needed to make appropriate health decisions*” [4,5]. However, broader definitions are gaining popularity and encompass a wider range of cognitive and social skills that enable people to feel empowered to take control and improve their health [6,7]. Nutbeam’s model of health literacy delineates three levels [7,8]:

1. Functional health literacy – basic reading comprehension and writing skills to understand health information/messages, together with knowledge of health conditions, services, and systems.

2. Communicative/interactive health literacy – higher level communicative and social skills required to extract and discuss information with others.

3. Critical health literacy skills – advanced literacy, cognitive, and social skills to analyze information and make informed decisions.

We propose that these levels correspond with the skills required to effectively use PtDAs and to engage in shared decision making activities: understanding health information, clarifying personal values, and communicating with health care providers.

A high proportion of adults have limited health literacy. In the U.S., estimates suggest that 36% (80 million adults) have “basic” or “below basic” health literacy [9]. Although methods of assessment vary, the picture is very similar in other developed countries [10]. The high levels of limited health literacy throughout the world provide a sharp reminder of the need to provide health information in a form that is appropriate for the literacy and numeracy levels of the majority of adults for whom it is prepared.

A person’s ability to effectively use a PtDA will be determined by both their health literacy skills *and* the quality and suitability of the PtDA [2]. Designers of PtDAs need to ensure that their tools can be accessed and understood by adults across the health literacy spectrum, including those with lower health literacy, and can support decisions that are both informed and behaviorally implemented.

## Theoretical justification for addressing health literacy in patient decision aids

We propose that Nutbeam’s multi-level model of health literacy provides a useful framework to help PtDA developers to address the needs of lower health literacy patients. Each of the levels of health literacy described in the model provides the skills required by patients to effectively use PtDAs and to engage in shared decision making. Individuals can obtain differential benefit from PtDAs depending on the relative differences in their health literacy. Those with the most basic functional health literacy will be able to obtain information from a PtDA and to use this as the basis for a more informed decision about their health. Those with higher level skills will be able to make greater use of available information to consider critically what is best for them, and to interact and communicate successfully with their health care provider in making a shared decision.

Attending to each of the levels of health literacy in PtDA design is important in order to meet the needs of patients with lower health literacy. This means developing PtDA materials to support consumers not only to read and understand evidence-based information (i.e., functional health literacy), but also to have the skills and confidence to communicate with health professionals and negotiate decisions (interactive/ communicative health literacy), and to clarify their values and think critically to make an informed decision (critical health literacy).

## Purpose and objectives

This paper systematically reviews the literature to guide PtDA developers as they attend to the health literacy needs of patients. We systematically reviewed empirical evidence relevant to health literacy and PtDAs with two principal aims:

I. To assess a) the effects of health literacy / numeracy on selected decision-making outcomes, and b) the effects of interventions designed to mitigate the influence of lower health literacy on decision-making outcomes, and

II. To assess the extent to which existing PtDAs a) account for health literacy, and b) are tested in lower health literacy populations.

## Empirical evidence: two reviews

### REVIEW I. Health literacy / health numeracy: the effects on decision-making outcomes and the effects of health literacy interventions

To address our first aim, we asked two review questions: 1) What is known about how health literacy affects decision-making outcomes, including knowledge, values clarity, and patient involvement? and 2) What is known about health literacy interventions’ ability to mitigate the effects of lower health literacy on decision-making outcomes?

### Review I: overview of methods

We searched two existing systematic reviews conducted for the U.S. Agency for Healthcare Research and Quality (AHRQ) in 2004 and 2011 on Health Literacy Interventions and Outcomes [1,11] for answers to these questions, and summarized their results if they were available. When questions were not answered (e.g., for outcomes and health literacy interventions related to values clarity or to patient involvement in decision making and communication (see below)), we performed our own systematic review and data synthesis.

### Review I: data sources and selection

To identify relevant articles, we searched articles included in the 2004 and 2011 systematic reviews performed for the AHRQ. These reviews had examined 1) the effects of health literacy (including numeracy) on health outcomes, and 2) the effects of interventions designed to mitigate the effects of low health literacy on health outcomes. These reviews had focused on English-language articles with an objective measure of health literacy that were published from 1966 to February 2011 and indexed in MEDLINE®, CINAHL, PsycINFO, ERIC, or the Cochrane Library database, and used the keywords literacy, numeracy, or terms or phrases related to measures thereof [1,11-13].

For our review, we also searched the titles and abstracts of articles that had been excluded from the 2011 AHRQ review [1] for the following reasons: 1) they didn’t use an objective measure of health literacy, or 2) they used an objective measure of health literacy, but reported only on outcomes that were not of interest to the AHRQ review (including outcomes related to clarifying values and participating in decision making). We did not search articles that had been excluded from the 2004 review [11] because they pre-dated landmark work in health literacy [4] and the first set of International Patient Decision Aid Standards (IPDAS) that called for values clarification and coaching as components of decision making [3].

To determine article inclusion, first one reviewer adhered to the inclusion criteria listed in Table 1, and excluded articles that were clearly unrelated to the questions guiding our review. Two reviewers then reviewed the remaining abstracts, and when necessary, reviewed full text articles to determine inclusion. Reviewers also reviewed the reference lists of included studies, prior narrative reviews on health literacy and decision making [2] or communication [14], and the bibliographies of systematic reviews on question-asking [15] and patient-centered care [16].

**Table 1:** Inclusion Criteria for Review I

Inclusion Category	Inclusion Criteria
Study Population	All ages, races, ethnicities, and cultural groups in developed countries. Health literacy and numeracy levels of individuals are either objectively or subjectively measured and reported for individuals in the population.

Health Outcomes	Includes decision-making outcome of interest: Values clarity Decision certainty Decision regret Decision confidence Desire for participation in decision Question asking Actual participation in decision Communication quality **•** Information provision/receipt **•** Good processes of communication/care **•** Satisfaction with communication/decision/care

Health Literacy Intervention	Interventions that authors report are specifically designed to mitigate the effects of low health literacy.

Study Design	Cross-sectional and cohort studies of the effects of health literacy and numeracy on decision making outcomes. Experimental studies of the effects of interventions on health outcomes.

Study Analyses	Stratified by Health Literacy Level with levels for analysis clearly specified.

Publication Status	Peer-reviewed articles. English language.

The inclusion criteria for this review mirrored the criteria used in the AHRQ reviews. However, our criteria were extended to include articles that included individual-level subjective assessments of health literacy, as well as outcomes related to clarifying values (e.g., decision uncertainty, decision regret, decision confidence, values clarity) and outcomes related to participating in decision making during the clinical encounter (e.g., patient activation, desire to participate, actual participation, communication quality). Our selection of these decision outcomes was guided by the general functional goals of PtDAs [17], which are: 1) To support users to understand health information relevant to their decision; 2) To support users to clarify their values; and 3) To support users to be actively involved in decision making and to communicate with others. Thus, these functional goals served as an overall conceptual structure for our review, as outlined in Table 2.

**Table 2:** Conceptual Structure for Review I: Three Function Goals of PtDAs

Function goals	Relevant literature included in first review
**PtDA Goal 1:** To support users to understand health information relevant to their decision.	a) Articles relating to knowledge or understanding of health information included in AHRQ reviews 2004 and 2011 [1,11]. b) Articles relating to interventions designed to improve these outcomes in individuals with low health literacy.

**PtDA Goal 2:** To support users to clarify their values.	a) Articles relating to patients' health values clarification, preference formation, uncertainty, decision satisfaction, decisional conflict and decisional regret. b) Articles relating to interventions designed to improve these outcomes in individuals with low health literacy.

**PtDA Goal 3:** To support users to be actively involved in decision making and to communicate with others.	a) Articles relating to patient involvement and preferences for involvement in decision making, patient activation, patient question asking, patient centered consultations and doctor-patient communication b) Articles relating to interventions designed to improve these outcomes in individuals with low literacy.

### Review I: data extraction and quality assessment

**Figure 1:**
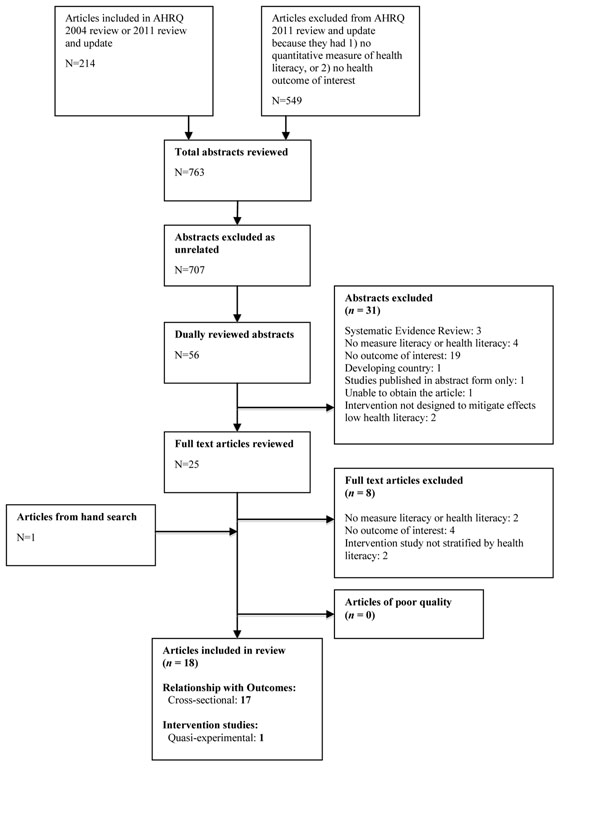
Flow Diagram for Inclusion / Exclusion of Articles in Review I

After inclusion was determined, a single reviewer entered information about studies into evidence tables, and a second reviewer checked abstractions for accuracy and consistency in presentation. Two reviewers independently rated study quality (good, fair, poor), using quality forms developed for the AHRQ reviews [1]. These forms assessed selection bias, measurement bias, confounding, and inadequate power. We excluded poor quality studies from our analysis and resolved disagreements about inclusion by consensus. Figure 1 is a flow diagram summarizing the full process of article exclusion / inclusion.

### Review I: data synthesis

Given the diversity in study outcomes and measurements, we synthesized data qualitatively and not by meta-analysis. We divided studies into those addressing values clarity and those addressing patient involvement and communication.

### Review I: results

Below, the results for the first review are structured according to three general functional goals of PtDAs [17]. Detailed summary tables for this first review appear in Appendices 1 to 4 (see Additional files 1, 2, 3, 4).

#### PtDA Goal 1: To support users to understand health information relevant to their decision

The two AHRQ reviews addressed our questions related to health literacy / numeracy, knowledge outcomes, and health literacy interventions. Their findings are summarized below, grouped according to our two review questions.

##### Relationship between health literacy / numeracy and the outcomes of knowledge, comprehension, and risk perception

The 2004 AHRQ review found that low health literacy (measured predominantly by the Rapid Estimate of Adult Literacy in Medicine (REALM) or Test of Functional Health Literacy in Adults (TOFHLA)) was related to patient knowledge in 14 of 16 studies dating from 1980 through 2003 [11]. Of the two studies that did not show a relationship, one was underpowered. Investigators concluded that the relationship was so clear that additional examination of this relationship was not necessary during a 2011 update.

The 2011 AHRQ review assessed the effect of numeracy on knowledge and on accuracy of risk perception from 1966 to February 2011 [1]. In four studies with a quantitative measure of numeracy, investigators found mixed effects of numeracy on general and disease-specific knowledge. Additionally, in five studies with a quantitative measure of numeracy, investigators found mixed effects of numeracy on the accuracy of perception of absolute risk and treatment benefit (expressed in multiple formats, including relative risk reduction (RRR), absolute risk reduction (ARR), or number needed to treat (NNT), with notable variations by task and measured outcomes (e.g., disparities in accuracy were even greater for participants when stating exact benefit rather than comparing the benefit of two treatments).

##### Effect of health literacy interventions designed to help low literacy individuals’ knowledge, comprehension, or accuracy of risk perception

A paper published from the 2011 AHRQ review that included an updated search [13] identified 38 studies published between 1966 and February 2011 that met the following criteria: they examined the effect of single or multiple literacy-directed strategies on knowledge or comprehension; they quantitatively assessed participants’ health literacy or numeracy; and they stratified analyses by health literacy level.

Fourteen of these studies (13 RCTs and 1 non-randomized controlled trial) specifically examined the effects of single strategies that might be useful in PtDA design. Among these 14 studies (which were reported in 12 articles) [18-29], investigators found multiple discrete design features that improved comprehension for low health literacy individuals in at least one study. These are summarized in Table 3. Of the remaining 24 studies that reported on interventions using a mixture of literacy-directed strategies, only one reported on a health literacy intervention in a PtDA context [30]. While this pre-post study of a prostate cancer PtDA reported improved knowledge among individuals in all health literacy subgroups (adequate literacy: + 1.27 points on a 10-point scale, adjusted *p* < 0.01; inadequate literacy +2.05 points, adjusted *p* < 0.01; *p* for interaction not reported), it did not describe its literacy-directed strategies in sufficient detail to allow recommendations to be derived.

**Table 3:** Partial Summary of Review I Findings Relative to PtDA Goal 1: to support users to understand health information relevant to their decision

Health Information Design Features that Improved Comprehension for Lower Health Literacy Individuals in at Least One Study
**•** Presenting essential information by itself or first [25] **•** Presenting numerical information in tables or pictographs rather than text [19,21,26] **•** Presenting numerical information so that the higher number is better (i.e. “nurses per patient”(more is better) rather than “patients per nurse” (less is better)) [25] **•** Presenting numerical information with the same denominator [21] **•** Using natural frequencies (e.g. 1 out of 100) to help individuals understand the probability of disease following testing [20] **•** Adding video to verbal narratives to improve the salience of information about health states [27]

#### PtDA Goal 2: To support users to clarify their values

In our own systematic review, we addressed the relationship between health literacy / numeracy and the outcome of values clarity, and the effect of health literacy interventions on values clarity.

##### Relationship between health literacy / numeracy and the outcome of values clarity

We found no studies that examined the relationship between health literacy and values clarity *per se*. Four cross-sectional studies [31] investigated the relationships between health literacy and decision uncertainty, decisional regret, and decision confidence. In the two studies that performed adjusted analyses, lower subjective health literacy increased decision uncertainty [31-34], and lower objective health literacy increased decision regret [32]. The effect of health literacy on decision confidence is less clear, with two unadjusted analyses showing mixed results [33,34]. See Table 4.

**Table 4:** Summary of Review I Findings Relative to PtDA Goal 2: to support values clarification among lower literacy consumers

Effect of health literacy on values clarification		
	No. of Studies	Summary of Findings

Decisional uncertainty Decisional regret	2 [31,32]	Lower health literacy associated with higher uncertainty and decisional regret.
Confidence in decision making	2 [33,34]	Effect unclear.
**Effect of health literacy intervention studies on values clarification**		

	No. of Studies	Summary of Findings

Decisional uncertainty	1 [35]	Video images to increase the salience of health states reduced decisional uncertainty, with strongest effect in lower health literacy patients.

##### Effect of health literacy interventions designed to help low literacy individuals clarify values

In the single intervention study in this group [35], Volandes et al. found that a PtDA using videos of patients to help elucidate the salience of various health states reduced decision uncertainty among all patients, with the greatest reduction found among patients with lower objective health literacy. See Table 4.

#### PtDA Goal 3: To support users to be actively involved in decision making and to communicate with others

In our own systematic review, we addressed the relationship between health literacy / numeracy and the outcomes of patient involvement and communication, and the effect of health literacy interventions on patient involvement and communication. See Table 5.

**Table 5:** Summary of Review I Findings Relative to PtDA Goal 3: to support patient involvement and communication among lower literacy consumers

Effect of health literacy on involvement and communication		
	No. of Studies	Summary of Findings

Preferences for participation in decision making / patient activation	3 [36-38]	Lower preference for involvement among lower health literacy patients.
	1 [39]	Patient activation associated with lower numeracy not health literacy.
Question asking	2 [40,41]	Less question asking among those with lower communicative HL. More clarification questions asked (indicating lack of understanding)
Level of involvement	2 [40,42]	Patients with lower communicative health literacy reported less involvement. Less mutuality observed between doctors and lower health literacy patients
Communication quality / Patient centered care	6 [36,43,44,46-48]	Less patient centered care among lower health literacy patients in 5 studies with adjusted analyses. 1 study reported effects varied by how numeracy was measured.
	3 [31,40,48]	Effects on communication quality (satisfaction and perceived quality) varied.
**Effect of health literacy intervention studies on involvement and communication**		

	No. of Studies	Summary of Findings

	None	Not applicable

##### Relationship between health literacy / numeracy and the outcomes of patient involvement and communication

Thirteen studies reviewed the relationship between health literacy level and various aspects of the decision-making encounter, such as patient activation and desire for participation, question-asking, broader participation, and communication quality. Three studies, including two that adjusted for confounders, reported that adults with lower health literacy were less likely to want to be involved in decision making compared to those with higher literacy [36-38]. A fourth showed that patient activation was associated with numeracy, but not health literacy in unadjusted correlational analysis [39].

Two papers addressed the relationship between literacy and question-asking [40,41]. One found that both level and type of subjective health literacy (functional, communicative, or critical) influenced question-asking, with lower levels of communicative health literacy associated with less question-asking [40]. The other found that lower objective health literacy patients were less likely to ask questions overall (although this just failed to reach statistical significance) [41] . Of the questions asked, lower health literacy patients asked significantly fewer medical and lifestyle questions and more clarification questions suggesting lack of understanding.

Two papers addressed patient involvement more broadly. One study reported patients with lower subjective “communicative” health literacy perceived they had less involvement in clinical consultations (although this just failed to achieve statistical significance, likely due to the small sample size) [40]. Arthur et al. reported a trend toward less “mutuality” (where both the physician and patient displayed a shared role in deciding the patient’s healthcare plan) among U.S. diabetes patients with limited objective health literacy [42].

Of the nine studies examining communication quality, four large high-quality studies with adjusted analyses reported lower patient-centered communication across several communication outcomes among those with lower objective health literacy (n = 2), lower subjective health literacy (n = 1), and lower subjective numeracy (n = 1). Communication was not specific to decision making, and the effect varied by outcome [43-46]. A fifth study, in which the sample overlaps with one of the previous four, found that ratings of patient-centered communication varied by measurement of numeracy. Patients with low objective numeracy reported more favorable communication and those with low subjective numeracy reported less favorable communication [47]. One unadjusted analysis found no difference between lower and higher health literacy patients’ perceptions that doctors facilitated their involvement in diabetes care [36].

Results of the remaining three studies varied by outcome. One study found that parents with lower objective health literacy reported more favorable interactions with their child’s healthcare provider [48]. Another found poorer decision satisfaction about breast cancer treatment among those with lower subjective health literacy [31]. Finally, one study found that those with lower critical health literacy were more likely to report they had received adequate information when more information was given (whereas those with higher critical literacy did not), offering a potential explanation for the contrasting findings reported above [40].

##### Effect of health literacy interventions designed to help low literacy individuals with communication

We identified no papers that tested health literacy interventions to assist the communication in the encounter, although one reported on the design of a cluster randomized trial to aid communication [49].

### Review II. Attending to health literacy among lower literacy populations in PtDA trials

To address our second aim, we asked the question: what level of attention is paid to health literacy in trials of PtDAs to date?

### Review II: overview of methods

To assess how health literacy has been addressed in PtDA trials, we systematically examined the PtDAs included in the published Cochrane Collaboration review of randomized controlled trials of PtDAs (including trials published to the end of 2009) [50]. We additionally updated their review with studies published to the end of 2010 (identified using the same search strategy).

### Review II: data sources

To identify articles, we used a set of 102 references maintained by the Cochrane Decision Aid group that included reports of 97 separate decision aids. This list is an up-to-date listing complete through to the end of 2010. We note that 16/102 papers published in 2010 and reporting on 11 decision aids were identified with the assistance of the Cochrane Group and added to the current review. These have not been included in published reports of the main Cochrane Collaboration’s systematic review of decision aids [50].

### Review II: data selection

After discussion among the 3 members of the review team, a set of review criteria was developed to indicate reading level accessibility to lower literacy audience and attention to literacy in the research design. The evaluative criteria for this review were based on those published in the 2006 IPDAS for literacy standards in PtDAs (see Table 6), with five new review criteria added to assess the level of attention paid to health literacy in the trials design (items 4-8 in Table 6).

**Table 6:** Eight Criteria used in Review II to Assess PtDAs’ Reading Level Accessibility and PtDA Trials’ Attention to Literacy

Original IPDAS Literacy Criteria	
1	Is the PtDA written at a level that can be understood by the majority of patients in the target group?
2	Is the PtDA written at a grade 8 or equivalent level or less according to readability score [SMOG or FRY]?
3	Does the PtDA provide ways to help patients understand information other than reading [audio, video, in-person]?
**PtDA Trial Design Criteria**	

4	Was the PtDA web-based?
5	Were study groups stratified by literacy?
6	Were study groups stratified by education?
7	Were low literacy groups over-sampled?
8	Were any conclusions drawn regarding literacy? (If yes, what?)

Each study was graded using these eight review criteria. Criteria were coded as present, absent, or unknown. Lack of indication of meeting any of the original IPDAS literacy criteria and lack of mention of health literacy at all were coded as “absent”. If articles made a global reference to the IPDAS literacy criteria, but made no specific mention of the particular IPDAS literacy criteria addressed, they were scored as “unknown”. See Table 7 for the working definitions of our full set of eight criteria and an explanation of how coding was performed. Additional detail about Review II’s criteria is available in Additional file 5 Appendix 5.

**Table 7:** Eight Criteria used in Review II to Assess PtDAs and PtDA Trials: Their Definitions and Coding Values

	Criterion Tag and Definition	Coding Value	Notes
***1***	***Lit Level Understood*** Is the PtDA written at a level that can be understood by a majority of patients in the study sample? If no mention of measuring comprehension was made a “2” was assigned. A simple increase in knowledge by the subjects was not taken to mean they understood.	Yes = 1 No = 2	

***2***	**<*Grade 9*** Was the PtDA stated to be at grade 9 readability level or less?	Yes = 1 No = 2 Don’t know = 9	If yes, what measure?

***3***	***Other than Text*** Does the PtDA provide ways to help patients understand information other than by reading? Includes specifically audio, video, web-based or reading aloud by researcher.	Yes = 1 No = 2 Don’t know = 9	If yes, what medium/media?

***4***	***Web-based*** The web is understood to mean access by computer, either at home or some other location with access to the world wide web. 1 = web-based and not all text; 2 = not web-based; 3 = web-based and all text; 9 = web-based and couldn’t tell whether all text-based or not. (If 2, then not web; if 1 or 3 or 9, then web-based.)	1 = multimedia web 2 = no web 3 = web text 9 = web but couldn’t tell content	

***5***	***Stratified by Literacy*** Was the study sample stratified by literacy level?	Yes = 1 No = 2 Don’t know = 9	If surrogate measure, what?

***6***	***Stratified by Education*** Was the study sample stratified by education level?	Yes = 1 No = 2 Don’t know = 9	If surrogate measure, what?

***7***	***Over –sampled Lower Literacy?*** Did the study over-sample lower literacy populations?	Yes = 1 No = 2 Don’t know = 9	

***8***	***Literacy Conclusion?*** Did the study draw literacy conclusions? (Code = 1 if any mention of literacy effects upon outcome variables, including if subgroup analyses were undertaken post-hoc)	Yes = 1 No = 2 Don’t know = 9	If yes, what?

We found that the original IPDAS literacy criterion #1 (“decision aid written at a level that can be understood by a majority of the sample”) to be very hard to determine. The intent of the criterion was that the PtDA developers should state the literacy requirements of the population for whom the PtDA was intended. The criterion was meant to encourage matching of population literacy requirements to the tool developed. However, in practice, the population literacy requirements were never stated, though sometimes the literacy level of the specific study sample was evaluated. To credit the attention paid by some investigators to the literacy levels of the sample, without having to attribute attention to population literacy requirements, we modified the wording of this criterion to code if literacy requirements of the subjects of the research were stated.

To test the functionality of our set of criteria, a 13% random sample was drawn from the pre-2010 set of 85 PtDAs, and assigned to 3 reviewers in groups of two in a blinded manner. One of us compared ratings and found that there was disagreement in 5.6% of the ratings. There were no obvious differences among the 3 reviewers in accuracy. We then discussed the differences and better specified the definitions.

### Review II: results

The total number of PtDA trials in the latest Cochrane update was 86. Eleven more trials were identified in the updated search through 2010, so that the total set of PtDA trials evaluated was 97 [50]. Table 8 lists the percentages and absolute numbers of individual studies that were evaluated according to our set of literacy criteria.

**Table 8:** Review II’s Summary of Attention Paid to Health Literacy in PtDA Trials

Criteria Used to Evaluate PtDA Trials % (n)								
	**Literacy Level Stated^Ϯ^**	**≤ Grade 8 Reading Age**	**Media other than Text^ϮϮ^**	**Web-Based**	**Stratified by Literacy**	**Stratified by Education**	**Lower Literacy Over-Sampled**	**Literacy Addressed in Conclusion**

Present	4 (4)	5 (5)	68 (67)	3 (3)	2 (2)	0 (0)	2 (2)	3 (3)
Absent	93 (91)	92 (89)	30 (29)	97 (94)	98 (95)	100 (97)	98 (95)	96 (94)
Unknown	1 (2)	3 (3)	1 (1)	0 (0)	0 (0)	0 (0)	0 (0)	0 (0)
Total	100%	100%	100%	100%	100%	100%	100%	100%
Trials	(97)	(97)	(97)	(97)	(97)	(97)	(97)	(97)

**^Ϯ^** *Most trials did not identify the literacy level of the intended audience. We operationalized this criterion to code “present” if the research sample group’s literacy requirements were stated.*

**^ϮϮ^***If the PtDA was text based*, *but included graphics of frequencies and other numeric data*, *these were considered to be text. This criterion was meant to provide for alternative media for non-readers such as pictures*, *video explanations of data*, *voice-over narration*, *etc.*

Five of the 97 RCTs reported the PtDA reading level as grade 8 or below [51-55]. Two trial reports stated that IPDAS literacy criteria were followed, but made no reference to which specific criteria [56,57]. Many PtDAs used media other than pure text, including interactive video and audio tape, although it was not clear whether these modalities were used specifically to address issues of literacy. There were eight PtDAs available on the web, though in many the presence or absence of content other than straight text could not be determined [58-66].

Seven studies recruited patients presumed to have lower health literacy (based on their educational status) and reported conclusions about health literacy [52,56,67-71]. Three randomized trials compared lower health literacy or education groups to higher health literacy groups. Two Australian studies developed PtDAs for bowel cancer screening[56,71], one specifically designed for a lower health literacy audience [56]. One U.S. study additionally addressed prostate cancer screening through a comparison “edutainment” PtDA and a paper PtDA [67]. All three of these PtDAs appear to have adhered to the IPDAS literacy criteria and included quantitative outcome data and explained technical terms. One PtDA was paper-based [71]; the remaining two used either audio or multi-media.

Two of three studies showed that PtDAs increased knowledge in samples presumed to have lower health literacy based on their educational status. Trevena et al. found a significant increase in knowledge in both lower- and highly-educated participants in the PtDA arm [71]. Smith et al. reported a 38% increase in knowledge and a 22% increase in informed choice (adequate knowledge and consistent attitudes and behavior) in lower education adults who received the literacy-sensitive PtDA compared with those who received standard information [56]. The third study—on prostate cancer screening—found no difference in knowledge between the “edutainment” and an audio-booklet presentation containing the same information [67]. However, the entertainment-based PtDA was associated with lower decisional conflict and greater self-advocacy when compared to the audio booklet among “low health literacy” patients. No differences between were observed for “high health literacy” patients.

## Discussion

Overall, our reviews suggest that patients with lower health literacy may be less able to use PtDAs effectively and to engage in shared decision making unless special attention has been paid to low health literacy in the PtDA development process. The reviews also indicate that patients with lower health literacy may be at greater need of support in decision making, given their higher levels of uncertainty and decisional regret, less involvement, and less patient-centered care. These findings suggest that attention to the needs of lower health literacy patients is required in PtDAs. Our reviews of the PtDA literature shows that health literacy has rarely been considered in the literature to date. However, in the small number of studies where the literacy needs of patients have been attended to, the results are encouraging.

Our reviews indicate that developers need to ensure they attend not only to issues of comprehension (functional health literacy), but also to values clarification and to the processes involved in the decision-making encounter itself (communicative and critical health literacy). If PtDAs are to be helpful in structuring and guiding decision making, they must address health literacy issues related to all of these processes. Because current evidence best supports improving the design and content of PtDAs—as summarized in Table 3—this should be a priority and will facilitate comprehension among lower health literacy patients.

The use of these strategies is recommended for the development of PtDAs for lower health literacy patients. However, intervention research relating to values clarification and the decision-making encounter are extremely limited, with almost no intervention studies available to guide PtDA developers. More research here is especially needed to understand potential differential effects of interventions by literacy level.

With regards to patient involvement, we note the consistent observation that patients with lower health literacy desire less involvement in decision making. We suggest that this may in part be a consequence of a lack of awareness that they can be involved and a lack of confidence in sharing the decision process with health care providers [72]. In patients with higher health literacy, desire for involvement has been found to increase when patients are shown the PtDA tools that are available [73]. Entwistle and colleagues emphasize the complexity of patients’ desire and capacity for involvement in health decisions and suggest that using a trusted source, such as a health care provider, to make decisions (i.e., “intellectual outsourcing”) can be an autonomy-enhancing strategy for some patients, including patients who do not feel they have the capacity to make a shared decision [74,75]. Entwistle highlights relational approaches to understanding health decision making that offer broader understandings of how patient autonomy in health decision making and in shared decision making can be achieved. Here, more research is needed to understanding the meaning and implications of involvement with lower literacy groups.

From our reviews of the PtDA literature, only 3 out of 97 PtDA trials included adults with lower education or health literacy, or used PtDAs designed explicitly to address lower health literacy audiences. In 90% or more of the trials, the reading needs of the participants and the reading level of the PtDAs were not reported. A clear bottom line is that PtDAs are rarely developed with lower literacy populations in mind, despite the observations that in many developed countries only half the population has more than basic reading skills [9], and that health information developed for adults at a grade 8 reading level is widely accepted by more educated as well as less educated users [76,77]. However, in PtDA studies in which education and health literacy were addressed, results across literacy groups are positive, with increases in knowledge and in informed choice reported.

In addition to the principles identified in these reviews, others’ reviews suggest strategies that may be useful when applied to PtDA development. For instance, we believe that particular principles based on the broader health literacy literature can be tentatively applied to the design, development, testing and implementation of PtDAs for lower health literacy patients [1]. See Table 9.

**Table 9:** Expert Opinion-Based Principles from the Broader Literature for Successful Health Literacy Interventions

Principles	**Rationale (based on broader health literacy literature) **[1]
Use high intensity interventions	Use multiple literacy-directed strategies to support knowledge acquisition and understanding. For example, design PtDAs using plain language, simple numbers, and a range of visual and linguistic techniques. Delivery of the PtDA requires multiple reinforcing contacts to support active decision-making.

Use theory-based interventions when appropriate	Theory can be used to maximize the impact of PtDAs. For instance, behavioral and communication theories applied in PtDAs can motivate engagement with the PtDA, or, if appropriate, engagement in specific behaviors.

Pilot test before full implementation	Pilot testing a PtDA involves examining the information needs and communication preferences of lower literacy populations, and examining the *whole* process of decision making among lower health literacy patients. This means checking not only understanding of the language and content, but also whether the PtDA helps users to clarify values, communicate with health professionals, and implement a decision.

Increased emphasis on skill building	PtDAs should be designed to help with skill building. This suggests that demonstrating and modeling values clarification and physician interactions in PtDAs may improve outcomes among low literacy users of PtDAs.

Delivery by a health professional	Deliver PtDAs by a health professional (e.g., pharmacist, health educator, nurse, physician) rather than by non-clinicians. This also suggests that delivery of PtDAs in the context of clinical care might result in the best outcomes.

Until further evidence is available, PtDA developers may also turn to best practices materials for development and testing that have been outlined by literacy experts and several national organizations (see, for example, [78-84]).

## Principles for PtDA development and areas for further research

The field must continue to push ahead with new research among lower health literacy populations. There are important deficits in the PtDA literature; much work is still needed to develop and test strategies to help adults with lower health literacy to gain key literacy skills, to engage in the values clarification process, and to be involved in doctor-patient communication. We propose that the most useful information in the field will result if PtDA developers do the following:

1. Use a measurable strategy to ensure that the language of the PtDA is written at a level that is understood by the majority of the target audience (e.g., Flesch-Kincaid, Simple Measure Of Gobbledegook (SMOG), Fry Readability Formula (or Fry Readability Graph), or other accepted approaches; see http://www.nlm.nih.gov/medlineplus/etr.html, Suitability Assessment of Materials (SAM) or Systemic Functional Linguistics [85].

2. State how the PtDA accommodated health literacy or numeracy requirements identified in the population and whether good health literacy principles were followed [e.g [78-83]].

3. Recruit, where possible, adequate numbers of low health literacy / numeracy individuals to evaluate effectiveness in this population. If full inclusion is not possible, report results of pilot studies among lower health literacy patients.

4. In conjunction with point 3, assess literacy / health literacy and/or numeracy levels in study samples by directly measuring functional health literacy and/or numeracy among a representative sample of the target sample of patients, using a recognized measure such as TOFHLA, REALM, or Newest Vital Signs (NVS).

5. Assess outcomes that have been shown to be or are postulated to be of specific concern for lower literacy patients.

Important research gaps in the field include a) the role of health literacy in the process of values clarification, b) the need to identify the characteristics of PtDAs that are universally acceptable and helpful, and c) the need to identify those characteristics that are particularly effective at enhancing shared decision making among people with lower health literacy/numeracy. Ultimately, this future research will contribute in important ways towards understanding the effects of PtDAs on health inequalities and towards ensuring that lower literacy/numeracy groups are not disadvantaged.

## Limitations

There are a number of limitations to our reviews of the decision-making literature. The decision for inclusion/exclusion was based on only a single assessment of each article. Although the articles closest in subject to our reviews’ purposes were assessed by two reviewers, it is possible that some relevant studies were missed. Other limitations include the inherent potential for publication bias, the diversity of measures used for similar outcomes, and the small number of physicians (despite adequate numbers of patients) that were included in studies of health literacy and communication. Indeed, many of the reviewed studies had small samples and larger studies are needed in future research. We also acknowledge there may be confounding and lack of control of relevant variables in some of the papers included in our reviews; however, we have presented the quality rating of our reviewed studies and indicated those with adjusted analyses. Although not a specific limitation of our reviews, one additional issue is that we excluded studies stratifying analyses by education. We made this choice because our search was not designed to pick up education as a proxy for literacy and we likely would have missed many relevant studies. It should be noted, however, that important information may be learned from such studies [67,86]. Furthermore, this field is growing quickly and we are already aware of new studies published since the study period [87,88]. While these studies support the conclusions in our reviews, it is possible that other new studies would provide new or different insights. Future updates will be important.

In our reviews of the PtDA literature, health literacy was not directly measured in any study. Proxy measures were used in three studies that reported having recruited individuals with unspecified deficits in education and health literacy. A limitation of our reviews is that we have not included the background articles that describe the development of PtDAs tested in the trials. Adding these may provide greater insight into how literacy was addressed. It does not, however, address the lack of attention to literacy in the trials and in identification of research questions. A related limitation is that we included only RCTs. Other studies of PtDAs that used different research designs may have included PtDAs that addressed low health literacy. We also note that there is now mixed evidence regarding the benefit of alternate media on improving outcomes for lower literacy patients, and so review criteria may need to be revised for future reviews [13].

## Conclusion

Because lower health literacy groups have lower levels of knowledge and involvement in health care, and some of the poorest health outcomes, they may be considered a priority group for better support in health decision making. Yet little attention has been given to this group in the PtDA literature. To enable more equitable access to PtDA resources and shared decision making, developers of PtDAs need to ensure that tools are accessible to lower as well as higher health literacy patients, and that lower health literacy groups are better equipped and supported to utilize PtDAs. Evidence is available to improve written information for lower health literacy patients; this evidence needs to be used more widely in PtDA development. However, there is little evidence to guide values clarification and increasing patient involvement in the consultation for this group of patients. More research is needed in these domains, in particular, to facilitate shared decision making among patients from varying health literacy backgrounds.

## List of abbreviations used

NVS: Newest Vital Signs; PtDA: patient decision aid; RCTs: randomized controlled trials; REALM: Rapid Estimate of Adult Literacy in Medicine; SAM: Suitability Assessment of Materials; SMOG: Simple Measure Of Gobbledegook; TOFHLA: Test of Functional Health Literacy in Adults

## Competing interests

Kirsten McCaffery and Marla Clayman have received research funding from the Informed Medical Decisions Foundation, a not-for-profit (501 (c)3) private foundation (http://www.informedmedicaldecisions.org). The Foundation develops content for patient education programs. The Foundation has an arrangement with a for-profit company, Health Dialog, to co-produce these programs. The programs are used as part of the decision support and disease management services Health Dialog provides to consumers through health care organizations and employers.

All other authors have no competing interests to declare.

## Authors’ contributions

KM and SLS drafted the manuscript with comments from MHR, DR, SKS and DN. All authors contributed to the drafting of the chapter on which the paper was based. SLS led the decision making review which involved MC, KM, SKS and MW. MHR and DR led the DA RCT review with input from KKB. DN, SKS and KM contributed to the theoretical framework for the paper. All authors read and approved the final manuscript.

## Supplementary Material

Additional file 1 **Appendix 1:** Review I: Overview of included studies Click here for file

Additional file 2 **Appendix 2:** Review I: Relationships between Health Literacy and Values Clarity/Decision Confidence Click here for file

Additional file 3 **Appendix 3:** Review I: Relationship between Health Literacy and Selected Patient-Physician Communication Variables Click here for file

Additional file 4 **Appendix 4:** Review I: Effect of Health Literacy Interventions on Decision-Making Outcomes Click here for file

Additional file 5 **Appendix 5:** Review II: Further Details about Procedures and Coding of PtDA Trials Click here for file

## Declarations

Publication of this supplement was partially funded by an unrestricted grant from the Informed Medical Decisions Foundation. The remaining costs were shared equally among the authors through a combination of institutional and personal funds. Administrative and editorial support for this supplement was also provided by The University of Texas MD Anderson Cancer Center.

This article has been published as part of *BMC Medical Informatics and Decision Making* Volume 13 Supplement 2, 2013: The International Patient Decision Aid Standards (IPDAS) Collaboration’s Quality Dimensions: Theoretical Rationales, Current Evidence, and Emerging Issues. The full contents of the supplement are available online at http://www.biomedcentral.com/bmcmedinformdecismak/supplements/13/S2.
