# Supplementary material for: Addressing health literacy in patient decision aids
Source: BMC Med Inform Decis Mak. 2013 Nov 29;13(Suppl 2):S10. doi: 10.1186/1472-6947-13-S2-S10 (PMC4042520; doi:10.1186/1472-6947-13-S2-S10)
Supplement: Additional file 1 — Appendix 1: Review I: Overview of included studies [file 1472-6947-13-S2-S10-S1.pdf]

Appendix 1.

Review I: Overview of Included Studies

Source	Design (Sample Size)	Population	Health Literacy or Educational Levels	Control	Intervention	Outcomes Category
**Relationship Between Literacy and Decision-Making Outcomes**						
Arthur et al., 2009 [1]	Cross-sectional (31 patients, 16 resident MDs)	African-American patients in 1 academic primary care office with a discussion about diabetes	45% < 6th grade on REALM	Not Applicable (N/A)	N/A	Level of Participation in Decision
Ciampa et al., 2010 [2]	Cross-sectional	3286 individuals who participated in the national Health Information Technology Survey	23% low objective numeracy by single item from Lipkus numeracy scale. NOTE: Subjective numeracy also assessed, but correlation poor with objective numeracy.	NA	NA	Communication Quality
Dewalt et al., 2007 [3]	Cross-sectional survey (268)	Type II diabetes patients attending a U.S. academic general internal medicine practice for a routine appointment	20% < 7th grade on REALM	N/A	N/A	Desire to Participate in Medical Decision Making, Communication Quality
Hawley et al., 2008 [4]	Cross-sectional (877)	Breast cancer patients from Los Angeles SEER area making a decision about surgical treatment	Subjective health literacy questions adapted from Chew, divided into tertiles of health literacy: 12% lowest tertile	N/A	N/A	Decision Regret, Communication Quality

Hibbard et al., 2007 [5]	Experimental design, although relationship of interest examined in cross-sectional fashion (303)	Community sample of employed-age adults making choices among hospitals	Mean score on objective measure adapted from Lipkus numeracy test = 9.3 (out of 15).<br><br>S-TOFHLA measured, but scores not reported.	N/A	N/A	Patient Activation
Ishikawa et al., 2009 [6]	Cross-sectional (134 pts; 4 MDs)	Diabetic outpatients attending outpatient University-affiliated hospital metabolic clinic in Japan	Subjective health literacy (HL) questions based on Nutbeam model.<br>Mean Functional HL: 3.4 (range 1.2-4)<br>Mean Communicative HL: 2.5 (range 1-4)<br>Mean Critical HL: 2.0 (range 1-3.5)	N/A	N/A	Question Asking, Participation in Decision Making, Communication Quality
Katz et al., 2007 [7]	Cross-sectional (57 pts; 21 MDs)	Patients attending primary care clinic in Atlanta, U.S.	37% $\leq$ 6th grade level on REALM	N/A	N/A	Questions Asking
Lillie et al., 2007 [8]	Cross sectional (163)	Stage I or II breast cancer patients at a University Breast Center making choices about adjuvant chemotherapy	Mean score on REALM = 63.<br>12% < 9th grade on REALM	N/A	N/A	Preference for Active Participation in Decision Making
Mancuso and Ricon, 2006 [9]	Cross-sectional survey (175)	English and Spanish speaking asthma patients attending a primary care practice in New York	Functional health literacy on TOFHLA:<br>Adequate: 82%<br>Marginal: 8%<br>Inadequate: 10%	N/A	N/A	Desire to Participate in Treatment Decisions

Martin et al., 2008 [10]	Cross-sectional (628)	628 patients from 5 community rheumatology clinics making a decision about rheumatoid arthritis treatment	Numeracy (4 questions) from DMARD. Knowledge profile: not reported.	N/A	N/A	Decision Confidence
Schillinger et al., 2004 [11]	Cross-sectional (408)	Diabetic patients in family medicine or general medicine clinics at one university hospital	Functional health literacy on S-TOFHLA: inadequate 38%, marginal 11%; adequate 51%	N/A	N/A	Communication Quality
Shone, 2009 [12]	Cross-sectional (499)	Parents and their asthmatic children in NY school district where 40% live in poverty	33% of parents with limited health literacy (<9th grade) on REALM.	N/A	N/A	Communication Quality
Smith et al., 2010 [13]	Cross-section-al (6024)	Individuals who participated in the national Health Information Technology Survey	37% with low subjective numeracy from single item about confidence with medical statistics.	N/A	N/A	Communication Quality
Sudore et al., 2009 [14]	Cross-sectional (771)	Primary care or cardiology patients	51% with limited (inadequate or marginal) health literacy on S-TOFHLA	N/A	N/A	Communication Quality
Sudore et al., 2010 [15]	Cross-sectional (205)	Multiethnic general medicine outpatients at a county hospital making a decision about life support	Functional health literacy on S-TOHFLA: Inadequate: 22% Marginal: 18% Adequate: 60%	N/A	N/A	Decision Uncertainty
Torres and Marks, 2009 [16]	Cross-sectional (106)	106 women recruited from a family clinic and making decisions about hormone replacement therapy	Functional health literacy on S-TOFHLA: Inadequate-46% Marginal-18% Adequate-36%	N/A	N/A	Decision Confidence
Wynia and	Cross-	Patients attending one of	57% had limited health	N/A	N/A	Communication quality

Osborn, 2010 [17]	sectional (2116)	13 heathcare settings (clinic or hospital)	literacy, averaging across 3 Chew subjective health literacy questions.			
**Effect of Health Literacy Interventions on Decision-Making Outcomes**						
Volandes et al., 2010 [18]	Quasi-exp. (pre-post) (146)	Patients attending urban and suburban primary care clinics in Boston (affiliated with teaching hospitals)	18% ≤ 6th grade on REALM<br><br>21% grade 7-8 on REALM<br>61% > 9th grade	N/A	A video of a patient with advanced dementia, depicting the important features of advanced dementia.	Decision Uncertainty

Abbreviations Used:

DMARD: Disease Modifying Anti-Rheumatic Drugs
HL: Health Literacy
MDs: Medical Doctors
REALM: Rapid Estimate of Adult Literacy in Medicine
SEER: Surveillance Epidemiology and End Results
S-TOFHLA: Short-Test of Functional Health Literacy in Adults
TOFHLA: Test of Functional Health Literacy in Adults

References

1. Arthur SA, Geiser HR, Arriola KRJ, Kripalani S: **Health literacy and control in the medical encounter: a mixed-methods analysis**. *J Natl Med Assoc* 2009, **101**:677-683.
2. Ciampa PJ, Osborn CY, Peterson NB, Rothman RL: **Patient numeracy, perceptions of provider communication, and colorectal cancer screening utilization**. *J Health Commun* 2010, **15**(Suppl 3):157-168.

3. DeWalt DA, Boone RS, Pignone MP: **Literacy and its relationship with self-efficacy, trust, and participation in medical decision making**. *Am J Health Behav* 2007, **31**:S27-S35.
4. Hawley ST, Janz NK, Hamilton A, Griggs JJ, Alderman AK, Mujahid M, Katz SJ: **Latina patient perspectives about informed treatment decision making for breast cancer**. *Patient Educ Couns* 2008, **73**:363-370.
5. Hibbard JH, Peters E, Dixon A, Tusler M: **Consumer competencies and the use of comparative quality information: it isn't just about literacy**. *Med Care Res Rev* 2007, **64**:379-394.
6. Ishikawa H, Yano E, Fujimori S, Kinoshita M, Yamanouchi T, Yoshikawa M, Yamazaki Y, Teramoto T: **Patient health literacy and patient–physician information exchange during a visit**. *Fam Prac* 2009, **26**:517-523.
7. Katz M, Jacobson T, Veledar E, Kripalani S: **Patient literacy and question-asking behavior during the medical encounter: a mixed-methods analysis**. *J Gen Intern Med* 2007, **22**:782-786.
8. Lillie SE, Brewer NT, O'Neill SC, Morrill EF, Dees EC, Carey LA, Rimer BK: **Retention and use of breast cancer recurrence risk information from genomic tests: the role of health literacy**. *Cancer Epidemiol Biomarkers Prev* 2007, **16**:249-255.
9. Mancuso CA, Rincon M: **Asthma patients' assessments of health care and medical decision making: the role of health literacy**. *J Asthma* 2006, **43**:41-44.
10. Martin RW, Head AJ, Rene J, Swartz TJ, Fiechtner JJ, McIntosh BA, Holmes-Rovner M: **Patient decision-making related to antirheumatic drugs in rheumatoid arthritis: the importance of patient trust of physician**. *J Rheumatol* 2008, **35**:618-624.
11. Schillinger D, Bindman A, Wang F, Stewart A, Piette J: **Functional health literacy and the quality of physician-patient communication among diabetes patients**. *Patient Educ Couns* 2004, **52**:315-323.
12. Shone LP, Conn KM, Sanders L, Halterman JS: **The role of parent health literacy among urban children with persistent asthma**. *Patient Educ Couns* 2009, **75**:368-375.
13. Smith SG, Wolf MS, von Wagner C: **Socioeconomic status, statistical confidence, and patient-provider communication: an analysis of the Health Information National Trends Survey (HINTS 2007)**. *J Health Commun* 2010, **15**(Suppl 3):169-185.

14. Sudore RL, Landefeld CS, Perez-Stable EJ, Bibbins-Domingo K, Williams BA, Schillinger D: **Unraveling the relationship between literacy, language proficiency, and patient-physician communication**. *Patient Educ Couns* 2009, **75**:398-402.
15. Sudore RL, Schillinger D, Knight SJ, Fried TR: **Uncertainty about advance care planning treatment preferences among diverse older adults**. *J Health Commun* 2010, **15**9(Suppl 2):159-171.
16. Torres RY, Marks R: **Relationships among health literacy, knowledge about hormone therapy, self-efficacy, and decision-making among postmenopausal health**. *J Health Commun* 2009, **14**:43-55.
17. Wynia MK, Osborn CY: **Health literacy and communication quality in health care organizations**. *J Health Commun* 2010, **15**:102-115.
18. Volandes AE, Barry MJ, Chang YC, Paasche-Orlow MK: **Improving decision making at the end of life with video images**. *Med Decis Making* 2010, **30**:29-34.
