# Supplementary material for: Addressing health literacy in patient decision aids
Source: BMC Med Inform Decis Mak. 2013 Nov 29;13(Suppl 2):S10. doi: 10.1186/1472-6947-13-S2-S10 (PMC4042520; doi:10.1186/1472-6947-13-S2-S10)
Supplement: Additional file 2 — Appendix 2: Review I: Relationships between Health Literacy and Values Clarity/Decision Confidence [file 1472-6947-13-S2-S10-S2.pdf]

Appendix 2.

Review I: Relationships between Health Literacy and Values Clarity/Decision Confidence

Authors, Year, Study Design, Sample Size	Population and Setting, Health Literacy or Education Level	Variables Used in Multivariate Analysis	Outcome Measure & Results By Health Literacy Skill Level	Differences in Results Between Health Literacy Skill Levels
Hawley et al., 2008 [1]<br>Cross-sectional<br>N = 877	Breast cancer patients from Los Angeles SEER registry making a decision about surgical treatment.<br><br>Subjective health literacy questions adapted from Chew: Lowest tertile -- 12%	Race/ethnicity<br>Age<br>Education<br>Marital status	Decision Regret<br><br>Not reported by literacy level	Decision Regret (low vs. high health literacy):<br>Odds ratio 3.5 (1.8 to 7.1)<br><br>Decision Regret (moderate vs. high health literacy):<br>Odds ratio 2.1 (1.4 to 3.1)
Martin, 2008 [2]<br>Cross-sectional<br>N = 628	Patients from 5 community rheumatology clinics making a decision about rheumatoid arthritis treatment.<br><br>Objective numeracy (4 questions) from DMARD Knowledge profile: Not reported	None	Decision Confidence measured by sub-index of COMRADE instrument<br><br>Not reported by numeracy level	Decision Confidence:<br>Pearson correlation 0.07; *p* not significant
Sudore, 2010 [3]<br>Cross-sectional<br>N = 205	Multiethnic general medicine outpatients at a county hospital	Age<br>Gender<br>Education	% participants reporting Decision Uncertainty on single question:	Adjusted odds ratio of reporting decision uncertainty (inadequate/marginal versus adequate): 2.11 (1.03 to 4.33)

	making a decision about life support<br><br>Functional health literacy on S-TOHFLA:<br>Inadequate: 22%<br>Marginal: 18%<br>Adequate: 60%	Health Status<br><br>Note: religiosity, trust, and prior experience not associated or included	Adequate health literacy: 34%<br>Marginal health literacy: 62%<br>Inadequate health literacy: 60%	
Torres, 2009 [4]<br>Cross-sectional<br>N = 106	106 women recruited from a family clinic and making decisions about hormone replacement therapy.<br><br>Functional health literacy on S-TOFHLA:<br>Inadequate: 46%<br>Marginal: 18%<br>Adequate: 36%	None	Mean Decision Confidence by 11-item decision self-efficacy scale	Decision Confidence:<br>Pearson correlation 0.70; $p < 0.01$

Abbreviations Used:

COMRADE: The Combined Outcome Measure for Risk Communication and Treatment Decision-Making Effectiveness
DMARD: Disease Modifying Anti-Rheumatic Drugs
SEER: Surveillance Epidemiology and End Results
S-TOFHLA: Short-Test of Functional Health Literacy in Adults

References

1. Hawley ST, Janz NK, Hamilton A, Griggs JJ, Alderman AK, Mujahid M, Katz SJ: **Latina patient perspectives about informed treatment decision making for breast cancer**. *Patient Educ Couns* 2008, **73**:363-370.

2. Martin RW, Head AJ, Rene J, Swartz TJ, Fiechtner JJ, McIntosh BA, Holmes-Rovner M: **Patient decision-making related to antirheumatic drugs in rheumatoid arthritis: the importance of patient trust of physician**. *J Rheumatol* 2008, **35**:618-624.
3. Sudore RL, Schillinger D, Knight SJ, Fried TR: **Uncertainty about advance care planning treatment preferences among diverse older adults**. *J Health Commun* 2010, **15**9(Suppl 2):159-171.
4. Torres RY, Marks R: **Relationships among health literacy, knowledge about hormone therapy, self-efficacy, and decision-making among postmenopausal health**. *J Health Commun* 2009, **14**:43-55.
