# Supplementary material for: Addressing health literacy in patient decision aids
Source: BMC Med Inform Decis Mak. 2013 Nov 29;13(Suppl 2):S10. doi: 10.1186/1472-6947-13-S2-S10 (PMC4042520; doi:10.1186/1472-6947-13-S2-S10)
Supplement: Additional file 3 — Appendix 3: Review I: Relationship between Health Literacy and Selected Patient-Physician Communication Variables [file 1472-6947-13-S2-S10-S3.pdf]

Appendix 3.

Review I: Relationship between Health Literacy and Selected Patient-Physician Communication Variables

Authors, Year, Study Design, Sample Size	Population and Setting, Health Literacy or Education Level	Variables Used in Multivariate Analysis	Outcome Measure<br><br>Results By Health Literacy Skill Level	Differences in Results Between Health Literacy Skill Levels
		**Selected Variable: Desire for Participation / Patient Activation**		
Dewalt et al., 2007 [1]<br>Cross-sectional survey<br>N = 268	Type I diabetes patients attending a U.S. academic general internal medicine practice for a routine appointment.<br><br>20% < 7th grade on REALM score.	None	Mean score for desire to participate in decision making, using Desire to Participate in Medical Decision Making Scale (range 0-100):<br><br>Low HL: 70<br>Higher HL: 78	Desire to participate in decision making (low versus high HL):<br>- 8 pts; $p < 0.001$.
Hibbard et al., 2007 [2]<br>Cross-sectional survey<br>N = 303	Community sample of employed-age adults making choices among hospitals.<br><br>Mean score on measure adapted from Lipkus objective numeracy test = 9.3 (out of 15).<br>S-TOFHLA measured, but not reported.	None	Mean patient activation measured by the Patient Activation Measure (range 0-100)<br><br>Individual scores by HL and numeracy level not reported.	Correlation between numeracy and patient activation: 0.16; $p < 0.01$<br><br>Correlation between HL and patient activation: 0.11; not significant<br><br>NOTE: Patient activation moderated the effects of low HL and numeracy on comprehension and “quality choice”
Lillie et al., 2007	Women with stage I or	Age	Preference for	Preference for participation in decision

[3]<br>Cross-sectional survey<br>N = 163	II breast cancer attending University of North Carolina Breast Centre making choices about adjuvant chemotherapy.<br><br>REALM mean score 63.6 (range 30-66)<br>≤3rd grade = 0%<br>4-6th grade = 2.5%<br>7-8th grade = 9.2%<br>High school = 88.3%<br><br>For analysis mean split used<br>Low ≤ 63<br>High > 63	Race<br>Marital status	participation in decision making<br><br>a) Decision to test<br><br>Higher HL:<br>58% active<br>39% shared decision making<br>3% passive<br><br>Lower HL:<br>41% active<br>47% shared decision making<br>12% passive<br><br>b) Decision on use of test results<br><br>Higher HL:<br>50% active,<br>45% shared decision making;<br>6% passive<br><br>Lower HL:<br>26% active,<br>40% shared decision making;<br>34% passive	making for two decisions (lower vs. higher HL):<br><br>a) Decision for testing:<br>Active: -17%<br>Shared decision making: +8%<br>Passive: 9%<br>Overall adjusted $p = 0.06$<br><br>b) Decision on use of test results<br>Active: -24%<br>Shared decision making: -5%<br>Passive: +28%<br>Overall Adjusted $p < 0.001$<br><br>NOTE: Larger adjusted effect of HL on participation preferences for decision on the use of test results (b) than on decision to test ($p = 0.003$)
Mancuso, 2006 [4]<br>Cross-sectional study	English and Spanish speaking asthma patients attending a	Gender<br>Race<br>Language	Desire to participate in treatment decisions using single question	Desire to participate in treatment decisions (marginal/inadequate vs. adequate HL):

N = 175	primary care practice in New York<br><br>Functional HL on TOFHLA:<br>Adequate: 82%<br>Marginal: 8%<br>Inadequate: 10%	Co-morbidity<br>Asthma duration<br>Asthma severity<br>Asthma control	Not reported by HL level	odds ratio 0.29 (0.13 to 0.65)

Selected Variable: Question Asking

Ishikawa et al., 2009 [5]<br><br>N = 134	Diabetic outpatients attending hospital metabolic clinic, Tokyo.<br><br>Mean Functional HL: 3.4 (range 1.2-4)<br>Mean Communicative HL: 2.5 (range 1-4)<br>Mean Critical HL: 2.0 (range 1-3.5)	Age<br>Gender<br>Education<br>Duration of diabetes<br>HbA1c<br>Visit length<br>Subjective measure: Functional HL; Communicative HL; Critical HL	Question Asking assessed by Roter Interactional Analysis System<br><br>Not reported by HL level	Question Asking (adjusted odds ratios)<br><br>Functional HL (higher vs. lower): 0.96 (0.75 to 1.24)<br><br>Communicative HL (higher vs. lower): 2.25 (1.76 to 2.88)<br><br>Critical HL (higher vs. lower): 1.24 (0.71 to 2.17)
Katz et al., 2007 [6]<br><br>N = 57	Patients attending primary care clinic, Grady Memorial Hospital, Atlanta, Georgia.<br><br>REALM<br>$\leq$ 6th grade level: 36.8% (N=21)	None	Mean number of total questions asked using Roter Interactional Analysis System<br><br>Mean number of questions asked in 11 content areas, including key medical content (therapeutic regimen, med. condition, lifestyle, request for services/meds) and bids for	Total questions asked:<br>Low vs. higher literacy: -3.1 questions, *p* = 0.07<br><br>Key medical content:<br>Low vs. higher literacy: -3 questions, *p* < .05<br><br>Bids for repetition:<br>Low vs. higher literacy: +0.5 questions, *p* < 0.05

			repetition<br><br>Total questions by HL level:<br>Low literacy: 10.2<br>Higher literacy: 13.3<br><br>Key medical content by HL level:<br>Low literacy: 5.7<br>High literacy: 8.7<br><br>Bids for repetition by HL level:<br>Low literacy: 0.7<br>High literacy: 0.2	NOTE: Other individual content area questions [including individual medical category questions (except lifestyle), psychosocial/feeling questions, procedural questions, understanding questions, reassurance questions, paraphrasing questions, and social questions] were not significantly different between literacy groups.

Selected Variable: Level of Involvement

Arthur et al., 2009 [7]<br>N = 31 (16 MDs)	African American patients with a discussion about diabetes<br><br>REALM<br>≤ 6th grade: 45% (n=14)	None	Observed Level of Participation by Taped Consultation and Roter Interactional Analysis System<br><br>≤6th grade:<br>Paternalistic 57%<br>Consumeristic 7%<br>Mutuality 36%<br><br>>6th grade:<br>Paternalistic: 23%<br>Consumeristic: 18%	Absolute Difference in Participation (Limited versus Adequate HL):<br><br>Paternalism: +34%*, unadjusted p = 0.06<br>Consumerism: -11%*<br>Mutuality: -17%*

			Mutuality 53%	
Ishikawa et al., 2009 [5]<br><br>N = 134 (4 MDs)	Diabetic outpatients attending hospital metabolic clinic, Tokyo.<br><br>Mean Functional HL: 3.4 (range 1.2-4)<br>Mean Communicative HL: 2.5 (range 1-4)<br>Mean Critical HL: 2.0 (range 1-3.5)	Age<br>Gender<br>Education<br>Duration of diabetes<br>HbA1c<br>Visit length<br>Subjective<br>Functional HL<br>Communicative HL<br>Critical HL	Patient perceived participation in consult on 5-item score.<br>Not reported by HL level.	Perceived Participation (beta coefficients):<br>Functional HL (higher vs. lower): β = -0.04; *p* = 0.93<br>Communicative HL (higher vs. lower): β = 1.63; *p* = 0.078<br>Critical HL (higher vs. lower): β = -0.42; *p* = 0.60<br><br>NOTE: Number of actual questions asked and information given was associated with perceived participation (β=0.07; *p* = 0.034).

Selected Variable: Communication Quality

Ciampa et al., 2010 [8]<br>Cross-sectional survey<br>N =3286	Individuals who participated in the national Health Information Technology Survey<br><br>23% low objective numeracy by single item from Lipkus numeracy scale	Age<br>Gender<br>Race<br>Income<br>Education<br>Health insurance<br><br>+/- 4 measures of perceived patient-provider communication	Patient Perception of Provider Communication using 4 questions from HINTS 2007<br><br>% reporting provider always allows health questions<br>Low Obj. Num.: 66%<br>High Obj. Num.: 58%<br><br>% reporting provider always pays attention to emotion:<br>Low Obj. Num.: 54%<br>High Obj. Num.: 40%	Adjusted OR for difference in good communication quality (low vs. high objective numeracy), 95% CI:<br><br>Health questions: 1.69 (1.07 to 2.68)<br><br>Attention to emotion: 1.71 (1.22 to 2.40)<br><br>Involves patients: 1.70 (1.13 to 2.56)<br><br>Ensures understanding: 1.54 (1.04 to 2.30)<br><br>NOTE: Those with low SUBJECTIVE numeracy had completely different results and reported that providers were LESS

			% reporting provider always involves patients in decision making:<br>Low Obj. Num: 59%<br>High Obj. Num: 47%<br><br>% reporting provider always ensures understanding:<br>Low Obj. Num: 65%<br>High Obj. Num: 54%	likely to allow questions, pay attention to emotion, involve patients, and ensure understanding.
Dewalt et al., 2007 [1]<br>Cross-sectional survey<br>N = 268	Type I diabetes patients attending a U.S. academic general internal medicine practice for a routine appointment.<br><br>20% < 7th grade on REALM score.	None	Mean patient perception that physician facilitated involvement using Facilitation of Patient Involvement Scale (range 0-100)<br><br>Low HL: 78<br>Higher HL: 75	Perceived facilitation of involvement (low vs. higher HL) (beta coefficient):<br><br>$\beta = +3\%$; $p = 0.15$
Ishikawa et al., 2009 [5]<br><br>N = 134	Diabetic outpatients attending hospital metabolic clinic, Tokyo<br><br>Mean Functional HL: 3.4 (range 1.2-4)<br><br>Mean Communicative HL: 2.5 (range 1-4)<br><br>Mean Critical HL: 2.0 (range 1-3.5)	Age<br>Gender<br>Education<br>Duration of diabetes<br>HbA1c<br>Visit length<br>Functional HL<br>Communicative HL<br>Critical HL	Patients' Perception of Whether Physician Provided Sufficient Information and Checked for Understanding (adapted from Process of Care In Diverse Populations Questionnaire)	Perceived Sufficient Information (beta coefficients):<br><br>Functional HL (higher vs. lower):<br>$\beta = -0.2$; $p = 0.30$<br>Communicative HL (higher vs. lower):<br>$\beta = -0.54$; $p = 0.403$<br>Critical HL (higher vs. lower):<br>$\beta = 1.64$; $p = 0.04$<br><br>NOTE: There was a 3-way interaction between communicative HL, actual

				information giving, and patients' perception of whether physicians provided sufficient information. Those with lower HL where more likely to report the physician provided sufficient information when more information was given. Perceived sufficiency of information did not vary by actual information provision for those with high HL.
Hawley et al., 2008 [9]<br>N = 877	Breast cancer patients from Los Angeles SEER registry making a decision about surgical treatment<br><br>Qualitative HL questions adapted from Chew:<br>Lowest tertile—12%	Race/ethnicity<br>Age<br>Education<br>Marital status	Decision Dissatisfaction<br>Not reported by literacy level	Decision Dissatisfaction (low vs. high HL):<br>Odds ratio 5.6 (2.9 to 11.1)<br><br>Decision Dissatisfaction (mod. vs. high HL):<br>Odds ratio 2.3 (1.6 to 3.3)
Schillinger et al., 2004 [10]<br>N = 408	Diabetes patients from two hospital- based clinics<br><br>38% inadequate HL on S-TOFHLA:	Age<br>Sex<br>Race/ethnicity<br>Education<br>Language<br>Insurance<br>Treatment regimen<br>HbA1c<br>Depression score<br>Duration of diabetes<br>Length of time in physician's care<br>Physician	% of poor Interpersonal Processes of Care, incl:<br><br>General clarity:<br>Inadeq.: 14%; Adeq.: 4%<br><br>Elicitation of Problems:<br>Inadeq.: 8%; Adeq.: 5%<br><br>Explanation of Condition:<br>Inadeq.: 9% Adeq.: 3%<br><br>Explanation of Care	Adjusted odds ratio poor process of care (inadequate versus adequate):<br><br>General Clarity: 6.29 (1.71 to 23.07)<br><br>Elicitation of Problems: 1.86 (0.54 to 6.36)<br><br>Explanation of Condition: 4.85 (1.2 to 19.58)<br><br>Explanation of Care Process: 2.7 (1.1 to1.66)

		assessment of Spanish fluency	Process:<br>Inadeq.: 21%; Adeq.: 10%<br><br>Explanation of Self-care:<br>Inadeq.: 16%; Adeq.: 18%<br><br>Empowerment:<br>Inadeq.: 21%; Adeq.: 12%<br><br>Decision making:<br>Inadeq.: 45%; Adeq.: 26%	Explanation of Self-care: 0.86 (0.39 to 1.89)<br><br>Empowerment:1.08 (0.38 to 3.06)<br><br>Decision Making: 1.66 (0.77 to 3.59)
Shone et al., 2009 [11]	Parents and their asthmatic children in NY school district where 40% live in poverty<br><br>33% of parents with limited HL (<9th grade) on REALM	Health insurance and<br>Parents' employment, ethnicity,<br>race, and<br>HL	Mean perceived Quality of Physician Interactions** (range NR, higher scores worse interaction)<br><br>Limited HL:<br>3.85<br>Adequate HL: 4.14	Adjusted beta-coefficient (Limited vs. Adequate HL): -0.20<br>(95% CI -0.3 to -0.1)
Smith et. Al., 2010 [12]<br><br>N = 6024	Individuals who participated in the national Health Information Technology Survey<br><br>37% with low subjective numeracy from single item about confidence with medical statistics	Age<br>Gender<br>Ethnicity<br>Education<br>Income<br>Marital status<br>Immigration<br>Regular health professional<br>Health insurance<br>Information preference<br>Survey mode	% reporting Poor Patient-Provider Interaction using 6 items from HINTS survey (range 6-24; 10-24= poor)<br><br>Low numeracy: 57%<br>High numeracy: 46%	Adjusted odds ratio poor Patient-Provider Interaction (vs. good interaction):<br><br>1.67 (1.43 to 1.96)

Sudore et al., 2009 [13]<br>N = 771	Patients from primary care and cardiology<br><br>51% Limited (Inadequate or Marginal) HL on S-TOFHLA	Age<br>Race<br>Gender<br>Education<br>Site<br>clustering by physician, language concordance	% poor communication by 3 questions approximating various communication types in the Interpersonal Processes of Care (IPC) questionnaire<br><br>Receptive communication:<br>Limited: 35%<br>Adequate: 24%<br><br>Proactive communication:<br>Limited: 35%<br>Proactive: 20%<br><br>Interactive communication:<br>Limited: 62%<br>Proactive: 50%	Adjusted odds ratio Poor Communication (Limited versus adequate HL):<br><br>Receptive communication:<br>1.95 (1.31 to 2.90)<br><br>Proactive communication:<br>1.82 (1.22 to 2.72)<br><br>Interactive communication:<br>1.40 (0.98 to 2.00)<br><br>NOTE: *p* for interaction by language concordance<br>= 0.10 for receptive communication;<br>= 0.38 for proactive communication;<br>= 0.23 for interactive communication.
Wynia and Osborn, 2010 [14]<br>N = 2116	Patients attending one of 13 healthcare settings (clinic or hospital)<br><br>57% with limited literacy, averaging across 3 questions from Chew et al.<br><br>For analysis, uses single literacy question: "difficulty understanding written information"	Gender<br>Age<br>Race/ethnicity<br>Primary language<br>Frequency of visiting hospital/clinic<br>Health care org type<br>HL	Mean score on composite rating of patient-physician communication drawn from the Organizational Communication Climate Assessment Toolkit (range 0-7)<br><br>Limited literacy: 5.2<br>Adequate literacy: 6.2	Mean difference in patient-physician communication (limited versus adequate literacy): -1, adjusted $p < 0.001$<br><br>In secondary analyses, those with limited HL had consistently lower odds of reporting doctors and nurses:<br>1) adequately explained things,<br>2) listened to them, and<br>3) made sure they understood them.<br>They were also less likely to report it was easy to ask questions and that the clinic communicated well with patients.

	NOTE: Results were similar for other HL questions; however, results were not statistically significant when HL defined by need to have someone help with reading hospital or clinic info.

*Calculated by research team
**Quality of interaction based on perceptions that providers understand how asthma affects their own child, that parents feel they are involved in decision making, and that parents feel free to contact the doctor with questions.

Abbreviations Used:
HINTS: Health Information Trends Survey
HL: Health Literacy
MDs: Medical Doctors
REALM: Rapid Estimate of Adult Literacy in Medicine
SEER: Surveillance Epidemiology and End Results
S-TOFHLA: Short-Test of Functional Health Literacy in Adults
TOFHLA: Test of Functional Health Literacy in Adults

References

1. DeWalt DA, Boone RS, Pignone MP: **Literacy and its relationship with self-efficacy, trust, and participation in medical decision making**. *Am J Health Behav* 2007, **31**:S27-S35.
2. Hibbard JH, Peters E, Dixon A, Tusler M: **Consumer competencies and the use of comparative quality information: it isn't just about literacy**. *Med Care Res Rev* 2007, **64**:379-394.
3. Lillie SE, Brewer NT, O'Neill SC, Morrill EF, Dees EC, Carey LA, Rimer BK: **Retention and use of breast cancer recurrence risk information from genomic tests: the role of health literacy**. *Cancer Epidemiol Biomarkers Prev* 2007, **16**:249-255.

4. Mancuso CA, Rincon M: **Asthma patients' assessments of health care and medical decision making: the role of health literacy**. *J Asthma* 2006, **43**:41-44.
5. Ishikawa H, Yano E, Fujimori S, Kinoshita M, Yamanouchi T, Yoshikawa M, Yamazaki Y, Teramoto T: **Patient health literacy and patient–physician information exchange during a visit**. *Fam Prac* 2009, **26**:517-523.
6. Katz M, Jacobson T, Veledar E, Kripalani S: **Patient literacy and question-asking behavior during the medical encounter: a mixed-methods analysis**. *J Gen Intern Med* 2007, **22**:782-786.
7. Arthur SA, Geiser HR, Arriola KRJ, Kripalani S: **Health literacy and control in the medical encounter: a mixed-methods analysis**. *J Natl Med Assoc* 2009, **101**:677-683.
8. Ciampa PJ, Osborn CY, Peterson NB, Rothman RL: **Patient numeracy, perceptions of provider communication, and colorectal cancer screening utilization**. *J Health Commun* 2010, **15**(Suppl 3):157-168.
9. Hawley ST, Janz NK, Hamilton A, Griggs JJ, Alderman AK, Mujahid M, Katz SJ: **Latina patient perspectives about informed treatment decision making for breast cancer**. *Patient Educ Couns* 2008, **73**:363-370.
10. Schillinger D, Bindman A, Wang F, Stewart A, Piette J: **Functional health literacy and the quality of physician-patient communication among diabetes patients**. *Patient Educ Couns* 2004, **52**:315-323.
11. Shone LP, Conn KM, Sanders L, Halterman JS: **The role of parent health literacy among urban children with persistent asthma**. *Patient Educ Couns* 2009, **75**:368-375.
12. Smith SG, Wolf MS, von Wagner C: **Socioeconomic status, statistical confidence, and patient-provider communication: an analysis of the Health Information National Trends Survey (HINTS 2007)**. *J Health Commun* 2010, **15**(Suppl 3):169-185.
13. Sudore RL, Landefeld CS, Perez-Stable EJ, Bibbins-Domingo K, Williams BA, Schillinger D: **Unraveling the relationship between literacy, language proficiency, and patient-physician communication**. *Patient Educ Couns* 2009, **75**:398-402.
14. Wynia MK, Osborn CY: **Health literacy and communication quality in health care organizations**. *J Health Commun* 2010, **15**:102-115.
