# Supplementary material for: Addressing health literacy in patient decision aids
Source: BMC Med Inform Decis Mak. 2013 Nov 29;13(Suppl 2):S10. doi: 10.1186/1472-6947-13-S2-S10 (PMC4042520; doi:10.1186/1472-6947-13-S2-S10)
Supplement: Additional file 4 — Appendix 4: Review I: Effect of Health Literacy Interventions on Decision-Making Outcomes [file 1472-6947-13-S2-S10-S4.pdf]

Appendix 4.

Review I: Effect of Health Literacy Interventions on Decision-Making Outcomes

Author, Date of Publication	Study Design	Control	Intervention	Sample Sizes	% Population Limited Literacy or Education	Outcomes	Difference
Volandes et al., 2010 [1]	Quasi-exper. (Pre-Post)	N/A	Video showing features of advanced dementia	146	18% ≤ 6th grade on REALM<br><br>21% grades 7-8 on REALM<br><br>61% > 9th grade	Mean uncertainty using Uncertainty subscale of Decisional Conflict Scale (range 3 to 15, with higher scores indicating less uncertainty)<br><br>Pre-intervention:<br>Low HL: 10.3<br>Marginal HL: 11.9<br>Adequate HL: 12.5<br><br>Post intervention:<br>Low HL: 13.9<br>Marginal HL: 14.4<br>Adequate HL: 14.6	Adjusted mean change in uncertainty (post-pre), 95% CI:<br><br>Low HL: + 3.5 (2.5-4.6)<br>Marginal HL: +2.5 (1.5-3.5)<br>Adequate HL: +2.0 (1.3-2.8)

Abbreviations Used:
HL: Health Literacy
REALM: Rapid Estimate of Adult Literacy in Medicine

References

1. Volandes AE, Barry MJ, Chang YC, Paasche-Orlow MK: **Improving decision making at the end of life with video images**. *Med Decis Making* 2010, **30**:29-34.
