# Supplementary material for: Addressing health literacy in patient decision aids
Source: BMC Med Inform Decis Mak. 2013 Nov 29;13(Suppl 2):S10. doi: 10.1186/1472-6947-13-S2-S10 (PMC4042520; doi:10.1186/1472-6947-13-S2-S10)
Supplement: Additional file 5 — Appendix 5: Review II: Further Details about Procedures and Coding of PtDA Trials [file 1472-6947-13-S2-S10-S5.pdf]

Appendix 5.

Review II: Further Details about Procedures and Coding of PtDA Trials

For Review Criterion 1 ("Lit Level Understood" = PtDA written at a level that can be understood by a majority of patients in the study sample), if no mention of determining comprehension was made, a value of "2" was assigned, indicating that comprehension was not evaluated. If a study tested an increase in knowledge from baseline, we did not infer that the meaning of the words in the PtDA was tested in the development.

Additionally, for Review Criterion 3 ("Other than Text" = the use of media other than text), the original criterion specifically calls for the use of pictures or other video explanations of data, voice-over narration, or the presence of a person to explain the material. In our review, if the PtDA was text-based, but included graphic representations of frequencies and other numeric data, these were considered to be text. This criterion was meant to provide for alternative media for non-readers, not a graphic representation of numbers, which are included in the definition of text.

For Review Criterion 5 ("Stratified by Literacy") and Review Criterion 6 ("Stratified by Education"), if only socio-education status was described but no stratification on these variables occurred, no credit was given for attention to low literacy in the study design. In every case, these measures were used to show similarity between control and experimental groups in order to control for literacy, not to address differences by literacy or education level.

If articles mentioned that they were developed using the IPDAS criteria, but did not provide any detail, this was problematic. There are many IPDAS criteria, and no developers indicated if all were followed. If the articles made no specific mention of literacy concerns or evaluation, but did indicate they followed the IPDAS criteria, these were scored as a "9", indicating unknown. Lack of indication of IPDAS criteria and lack of mention of literacy at all was scored "2", indicating absent.

We then iteratively performed sample analyses and discussed details of the Review Criteria until all agreed. Data analysis was descriptive, showing proportion of studies meeting each Review Criterion.
